# Molecular characterization and expression of six heat shock protein genes in relation to development and temperature in *Trichogramma chilonis*

**DOI:** 10.1371/journal.pone.0203904

**Published:** 2018-09-18

**Authors:** Jiequn Yi, Han Wu, Jianbai Liu, Xueshuang Lai, Jixing Guo, Dunsong Li, Guren Zhang

**Affiliations:** 1 State Key Laboratory for Biocontrol & Institute of Entomology, Sun Yat-Sen University, Guangzhou, Guangdong, China; 2 Guangdong Provincial Key Laboratory of High Technology for Plant Protection/Plant Protection Research Institute, Guangdong Academy of Agricultural Sciences, Guangzhou, Guangdong, China; Université de Genève, SWITZERLAND

## Abstract

*Trichogramma* is a kind of egg parasitoid wasp that is widely used to control lepidopterous pests. Temperature is one of the main factors that determines the various life activities of this species, including development, reproduction and parasitism efficiency. Heat shock proteins (HSPs) are highly conserved and ubiquitous proteins that are best known for their responsiveness to temperature and other stresses. To explore the potential role of HSPs in *Trichogramma* species, we obtained the full-length cDNAs of six HSP genes (*Tchsp10*, *Tchsp21*.*6*, *Tchsp60*, *Tchsp70*, *Tchsc70-3*, and *Tchsp90*) from *T*. *chilonis* and analyzed their expression patterns during development and exposure to temperature stress. The deduced amino acid sequences of these HSP genes contained the typical signatures of their corresponding protein family and showed high homology to their counterparts in other species. The expression levels of *Tchsp10*, *Tchsp21*.*6* and *Tchsp60* decreased during development. However, the expression of *Tchsc70-3* increased from the pupal stage to the adult stage. *Tchsp70* and *Tchsp90* exhibited the highest expression levels in the adult stage. The expression of six *Tchsps* was dramatically upregulated after 1 h of exposure to 32 and 40°C but did not significantly change after 1 h of exposure to 10 and 17°C. This result indicated that heat stress, rather than cold stress, induced the expression of HSP genes. Furthermore, the expression of these genes was time dependent, and the expression of each gene reached its peak after 1 h of heat exposure (40°C). *Tchsp10* and *Tchsp70* exhibited a low-intensity cold response after 4 and 8 h of exposure to 10°C, respectively, but the other genes did not respond to cold at any time points. These results suggested that HSPs may play different roles in the development of this organism and in its response to temperature stress.

## Introduction

Wasps of the genus *Trichogramma* (Hymenoptera: Trichogrammatidae) are tiny egg parasitoids of numerous insect species that are distributed around the world [1–3]. These wasps are easily mass reared and have a broad range of hosts [4]. Since 1975, several species of *Trichogramma* have been commonly used as biological control agents for various pests in agricultural and forest systems [5, 6]. Among these parasitoid wasps, *T*. *chilonis* is one of the most successful species in controlling lepidopterous pests, including *Chilo* spp. (Lepidoptera: Pyralidae), *Helicoverpa armigera* (Lepidoptera: Noctuidae) and *Pectinophora gossypiella* (Lepidoptera: Gelechiidae) [2]. In China, *T*. *chilonis* is widely distributed and is employed in integrated pest management for rice, cotton, sugarcane and other crops [7].

Temperature is a vital factor that determines the distribution and abundance of animals [8]. It is also crucial for the successful introduction of *Trichogramma* species because it influences their development, survival, reproduction, and sex ratio, as well as their parasitism efficiency [3, 9]. Previous studies have claimed that *Trichogramma* species can live under a wide range of temperatures from 9 to 36°C [10]. The temperature range of 25–30°C is considered optimal for rearing *T*. *chilonis* in the laboratory [11]. Temperatures beyond the optimal conditions could cause detrimental effects on various biological aspects of the wasps. For instance, *T*. *chilonis* and three other *Trichogramma* species cannot parasitize the eggs of *Cnaphalocrocis medinalis* (Guene´e) (Lepidoptera: Pyralidae) at 36°C [12]. The emergence and host parasitization of *T*. *chilonis* are 98.0% and 95.6% at 28°C but decrease to 33.7% and 60.1%, respectively, at 35°C [9]. Moreover, a low temperature of 15°C leads to long developmental periods for *T*. *chilonis* (26.3 days) and *Trichogrammatoidea bactrae* (25.6 days) (Hymenoptera: Trichogrammatidae) [4]. Although detrimental consequences caused by temperature stress have been well reported, little is known about the molecular response to temperature stress in *Trichogramma*.

Heat shock proteins (HSPs) are highly conserved and ubiquitous proteins that are best known for their responsiveness to multiple stresses such as extreme temperatures, desiccation, anoxia, hypertonic stress, ultraviolet radiation, heavy metals, ethanol, and other contaminants [13, 14]. In general, HSPs act as molecular chaperones that promote correct refolding of proteins and prevent the misfolding or aggregation of proteins [15]. According to their molecular weight and homology, HSPs are classified into several families, including HSP100, HSP90, HSP70, HSP60, HSP40 and small HSPs (sHSPs) [16]. To data, certain groups of HSP genes (*hsps*) have been identified and cloned from various insects such as *Spodoptera litura* (Lepidoptera: Noctuidae), *Thitarodes pui* (Lepidoptera: Hepialidae), *Leptinotarsa decemlineata* (Coleoptera: Chrysomelidae) and *C*. *suppressalis* [17–20]. An increasing number of *hsps* have been shown to respond to temperature stress [17, 21, 22]. In an endoparasitoid wasp, *Pteromalus puparum* (Hymenoptera: Pteromalidae), six *hsps* are induced by 1 h of exposure to −3 and 36°C, including *hsp20*, *hsp40*, *hsp60*, *hsp70*, *hsc70*, and *hsp90* [23]. The expression patterns of five *hsps* vary but are indeed induced by heat or cold stress in *Cotesia chilonis* (Hymenoptera: Braconidae) [24]. In addition, *hsps* have also been reported to be involved in the developmental processes of endoparasitoid wasps such as *Venturia canescens* (Hymenoptera: Ichneumonidae), *Macrocentrus cingulum* (Hymenoptera: Braconidae) and *C*. *vestalis* [25–27].

*Trichogramma* wasps are often released in the fields during pupal stage [5]. Various biological parameters of the released parasitoids are influenced by ambient temperature [3, 9]. To data, the molecular mechanism of thermal tolerance remains unclear. In present study, six *hsps* of *T*. *chilonis* (*Tchsp10*, *Tchsp21*.*6*, *Tchsp60*, *Tchsp70*, *Tchsc70-3*, and *Tchsp90*) were cloned and characterized, and their expression profiles during development were explored. In addition, individuals at the pupal stage were collected to explore the expression patterns of these six *hsps* in response to various levels of temperature stress (10, 17, 32 and 40°C for 1 h). The temporal expression patterns of six *Tchsps* were also investigated during cold (10°C) and heat (40°C) exposure. To our knowledge, this is the first report on the isolation and analysis of *hsps* from *T*. *chilonis*. Our results are expected to help elucidate the potential contribution of these HSPs to thermal tolerance and development.

## Materials and methods

### Insects

Prepupae of *T*. *chilonis* (parasitized eggs) and eggs of *Corcyra cephalonica* (Stainton) (Lepidoptera: Pyralidae) were obtained from the Plant Protection Research Institute, Guangdong Academy of Agricultural Sciences, People’s Republic of China. *T*. *chilonis* cultures were maintained on irradiated *C*. *cephalonica* eggs for several generations at 25 ± 1°C with 75 ± 5% relative humidity and a 14 L:10 D photoperiod.

### Sampling at different developmental stages

The irradiated eggs of *C*. *cephalonica* were glued on 4 paper cards (2 × 1 cm) and exposed to freshly emerged *T*. *chilonis* for 30 min. These egg cards were transferred to different glass cylinders and maintained at 25 ± 1°C with 75 ± 5% relative humidity and a 14 L:10 D photoperiod. Parasitized eggs on different cards were dissected to collect the larvae, prepupae, pupae and adults of *T*. *chilonis*. The developmental stages of *T*. *chilonis* were confirmed under a stereoscope as described in previous studies [28, 29]. Every 6 h, a small number of parasitized eggs were dissected to determine the developmental stage of *T*. *chilonis*. At the larval stage of *T*. *chilonis*, the colors of individuals and parasitized eggs were both white. Larvae with oval shapes were collected. At the prepupal stage, the color of parasitized eggs was black, and pulm spots were visible on the body. Prepupae were collected when pulm spots disappeared from the head and tail. At the pupal stage, the color of parasitized eggs turned deep, red compound eyes appeared on the body, and pulm spots disappeared. Pupae with small black spots on their bodies were collected. The adults were collected once they emerged from the eggs. To collect corresponding individuals, parasitized eggs were immediately placed on a filter-paper soaked with Sample Protector for RNA/DNA (TaKaRa, Dalian, China) and dissected under a stereoscope. The specimens were immediately transferred to TRIzol (Invitrogen, Darmstadt, Germany) and stored in a -80°C refrigerator. Fifty wasps from each developmental stage were collected. The experiment was repeated three times.

### Temperature exposure

Considering that *T*. *chilonis* wasps are often released as pupae inside the host eggs, these parasitized eggs were chosen for temperature exposure experiments. The parasitized eggs were exposed to temperatures of 10, 17, 32 and 40°C for 1 h, and parasitized eggs kept at 25°C were collected as controls. These parasitized eggs were then dissected to collect wasps under a stereoscope. In addition, wasps were collected at different time points (1, 2, 4 and 8 h) during cold (10°C) and heat (40°C) exposure. The sampling method was the same as described above.

### Cloning the full-length cDNA of *hsps*

Total RNA from adults was isolated with a TRIzol Reagent Kit according to the supplier’s instructions. Assessment of the quality and quantity of total RNA was performed by electrophoresis and with a NanoDrop 2000 (Thermo Fisher Scientific, Wilmington, DE, USA). First-strand cDNA was generated with a PrimeScript^TM^ RT Reagent Kit (TaKaRa, Dalian, China). Templates for 5’ and 3’ RACE were constructed using a SMART^TM^ RACE cDNA Amplification Kit (Clontech, California, USA). Primers (S1 Table) were designed based on the nucleotide sequences from the transcriptome data of *T*. *chilonis* (SRA accession number: SRP119024). PCR products were cloned and then sequenced by Sangon (Shanghai).

### Bioinformatics analysis

Using the DNASTAR software package, full-length cDNAs of *hsps* were obtained based on the sequenced fragments. The BLAST search was performed to find homologous sequences in GenBank. Multiple sequence alignment and identity analysis were performed using DNAMAN software. The open reading frame (ORF) and deduced amino acid sequence of each *hsp* were identified and obtained using ORF Finder (http://www.ncbi.nlm.nih.gov/gorf/gorf.html). The predicted molecular weight and theoretical isoelectric point (pI) of the deduced proteins were predicted with the ExPASy (http://www.expasy.org/). Domains were predicted by SMART tool (http://smart.embl-heidelberg.de/). Phylogenetic analysis was performed using MEGA software (version 6.0) with the 1000 bootstrap replicates. Five neighbor-joining (NJ) phylogenetic trees were constructed using members of HSP10, sHSPs, HSP60, HSP70 and HSP90 familiy.

### Quantitative real-time PCR

Total RNA of the samples from each treatment was extracted and reverse transcribed as described above. Primers were designed based on the conserved regions of the *hsps*, and glyceraldehyde-3-phosphate dehydrogenase (*gapdh*) was used as the control (S1 Table). The real-time PCR reaction was performed in a 10 μL reaction volume following the manufacturer’s protocol for SYBR ® Premix Ex Taq™ (TaKaRa, Dalian, China). The expression profiles of *hsps* were determined on a Roche 480 Real-Time PCR System (Roche, Switzerland) under the following conditions: 95°C for 3 min, 40 cycles of 95°C for 10 s, 60°C for 20 s and 72°C for 20 s. The melting curve analysis was applied to ensure the specificity of primers at the end of the program. The relative abundance of each *hsp* was calculated according to the 2^−ΔΔCt^ method [30].

### Statistics

The expression values of the *hsps* are presented as the means ± SEM. Statistical analysis was performed by SPSS v.16.0 software (SPSS, Chicago, IL, USA) with one-way analysis of variance (ANOVA) and Duncan’s post hoc tests.

## Results

### Characterization of *hsp* genes

#### *Tchsp10*.

**Fig 1:**
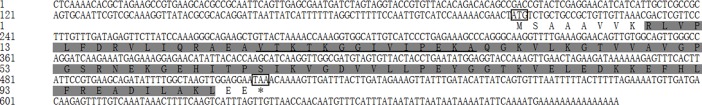
Nucleotide sequence and deduced amino acid sequence of *Tchsp10*. The initiation and stop codons are marked with boxes. The conserved domain is shaded in light gray. The mobile loop is underlined.

The full-length cDNA of *Tchsp10* was 705 bp, including an ORF of 315 bp, a 5’- untranslated region (UTR) of 205 bp and a 3’-UTR of 185 bp (GenBank accession number MH490973). The ORF of *Tchsp10* encoded a polypeptide of 104 amino acids with a predicted molecular weight of 11.26 kDa and a pI of 8.93. TcHSP10 showed topical Cpn10 superfamily characteristics with a conserved domain (aa 9–102) and and a mobile loop (aa 25–38) (Fig 1).

#### *Tchsp21*.*6*.

**Fig 2:**
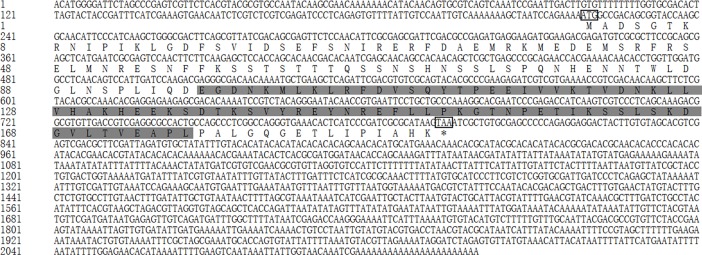
Nucleotide sequence and deduced amino acid sequence of *Tchsp21*.*6*. The initiation and stop codons are marked with boxes. The α-crystallin domain (ACD) is shaded in light gray.

The full-length cDNA of *Tchsp21*.*6* was 2119 bp, including an ORF of 576 bp, a 5’- UTR of 218 bp and a 3’-UTR of 1325 bp (GenBank accession number MH490974). The ORF of *Tchsp21*.*6* encoded a polypeptide of 191 amino acids with a predicted molecular weight of 21.68 kDa and a pI of 5.6. TcHSP21.6 was a typical small HSP, containing a metazoan α-crystalline domain (ACD) (Fig 2).

#### Tchsp60

**Fig 3:**
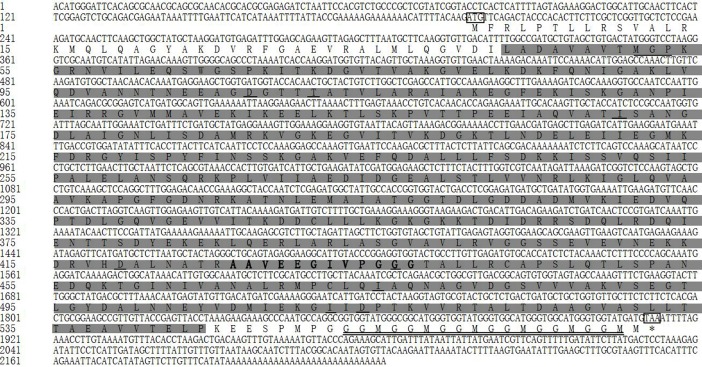
Nucleotide sequence and deduced amino acid sequence of *Tchsp60*. The initiation and stop codons are marked with boxes. The conserved domain is shaded in light gray. The ATP binding sites are underlined. The GGM repeat motif is marked with a double line. The classical mitochondrial HSP60 signature motif is shown in bold.

The full-length cDNA of *Tchsp60* was 2222 bp, including an ORF of 1716 bp, a 5’-UTR of 197 bp and a 3’-UTR of 309 bp (GenBank accession number MH490975). The ORF of *Tchsp60* encoded a polypeptide of 571 amino acids with a predicted molecular weight of 60.48 kDa and a pI of 5.18. TcHSP60 contained a classical mitochondrial HSP60 signature motif (AAVEEGIVPGGG), a C-terminal Gly-Gly-Met repeat (GGM repeat motif) and ATP/ADP binding sites (Fig 3).

#### Two TcHSP70 genes

The full-length cDNA of *Tchsp70* was 2573 bp, including an ORF of 1992 bp, a 5’-UTR of 13 bp and a 3’-UTR of 568 bp (GenBank accession number MH490976). The ORF of *Tchsp70* encoded a polypeptide of 663 amino acids with a predicted molecular weight of 73.26 kDa and a pI of 5.62.

The full-length cDNA of *Tchsc70-3* was 2668 bp, including an ORF of 1992 bp, a 5’-UTR of 186 bp and a 3’-UTR of 490 bp (GenBank accession number MH490977). The ORF of *Tchsc70-3* encoded a polypeptide of 663 amino acids with a predicted molecular weight of 73.34 kDa and a pI of 5.12.

**Fig 4:**
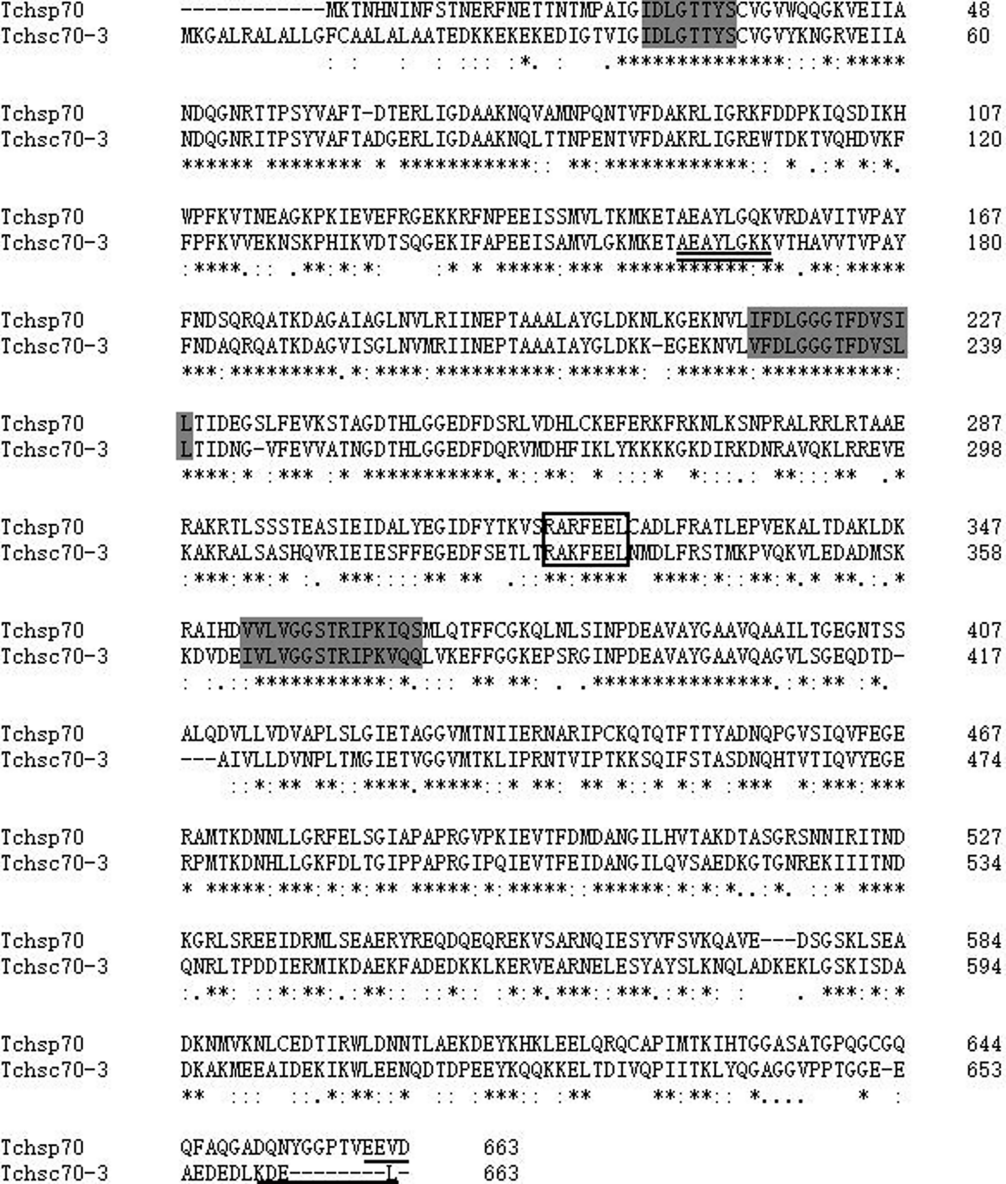
Multiple amino acid sequence alignments of two genes in the HSP70 family. Three signature motifs of the HSP70 family are shown in light gray. The non-organellar consensus motif is boxed, the localization motif is underlined, and the ATP/GTP binding site is double underlined.

The two TcHSP70 sequences contained three conserved signatures, an ATP-GTP binding site and a non-organellar consensus motif (Fig 4). In addition, the KDEL motif was identified in the deduced amino acid sequence of *Tchsc70-3*. The EEVD motif was found at the C-terminus of TcHSP70.

#### Tchsp90

The full-length cDNA of *Tchsp90* was 2643 bp, including an ORF of 2181 bp, a 5’-UTR of 145 bp and a 3’-UTR of 317 bp (GenBank accession number MH490980). The ORF of *Tchsp90* encoded a polypeptide of 726 amino acids with a predicted molecular weight of 83.48 kDa and a pI of 4.88.

**Fig 5:**
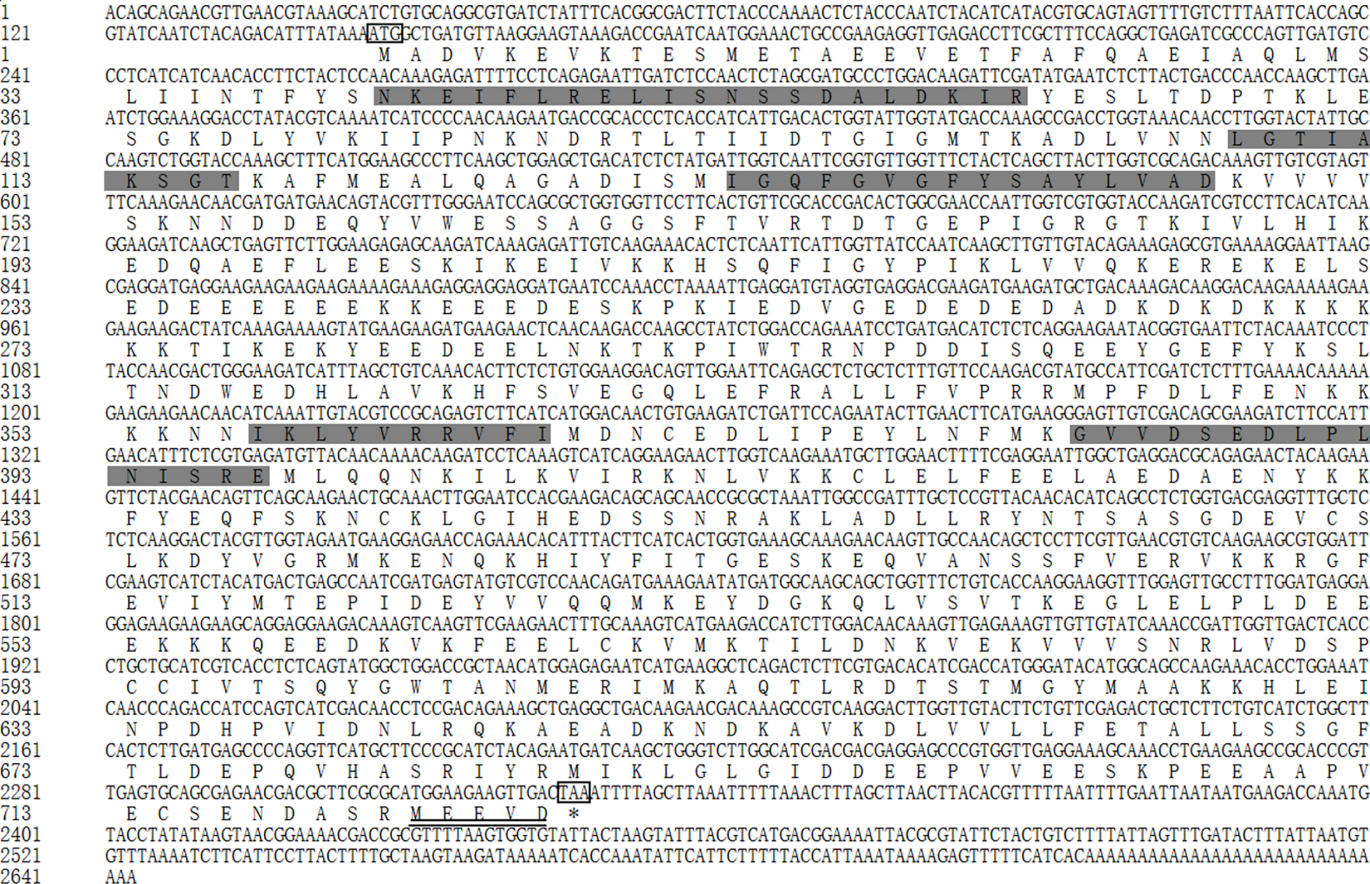
Nucleotide sequence and deduced amino acid sequence of *Tchsp90*. The initiation and stop codons are marked with boxes. Five signature motifs of the HSP90 family are shown in light gray. The localization motif is double underlined.

Five highly conserved signature sequences of the HSP90 family were found, including NKEIFLRELISNSSDALDKIR (aa 41–61), LGTIAKSGT (aa 108–116), IGQFGVGFYSAYLVAD (aa 132–147), IKLYVRRVFI (aa 357–366) and GVVDSEDLPLNISRE (aa 383–397) (Fig 5). The MEEVD motif was identified at the C-terminus of the deduced amino acid sequence.

#### Phylogenetic analysis of TcHsps

**Fig 6:**
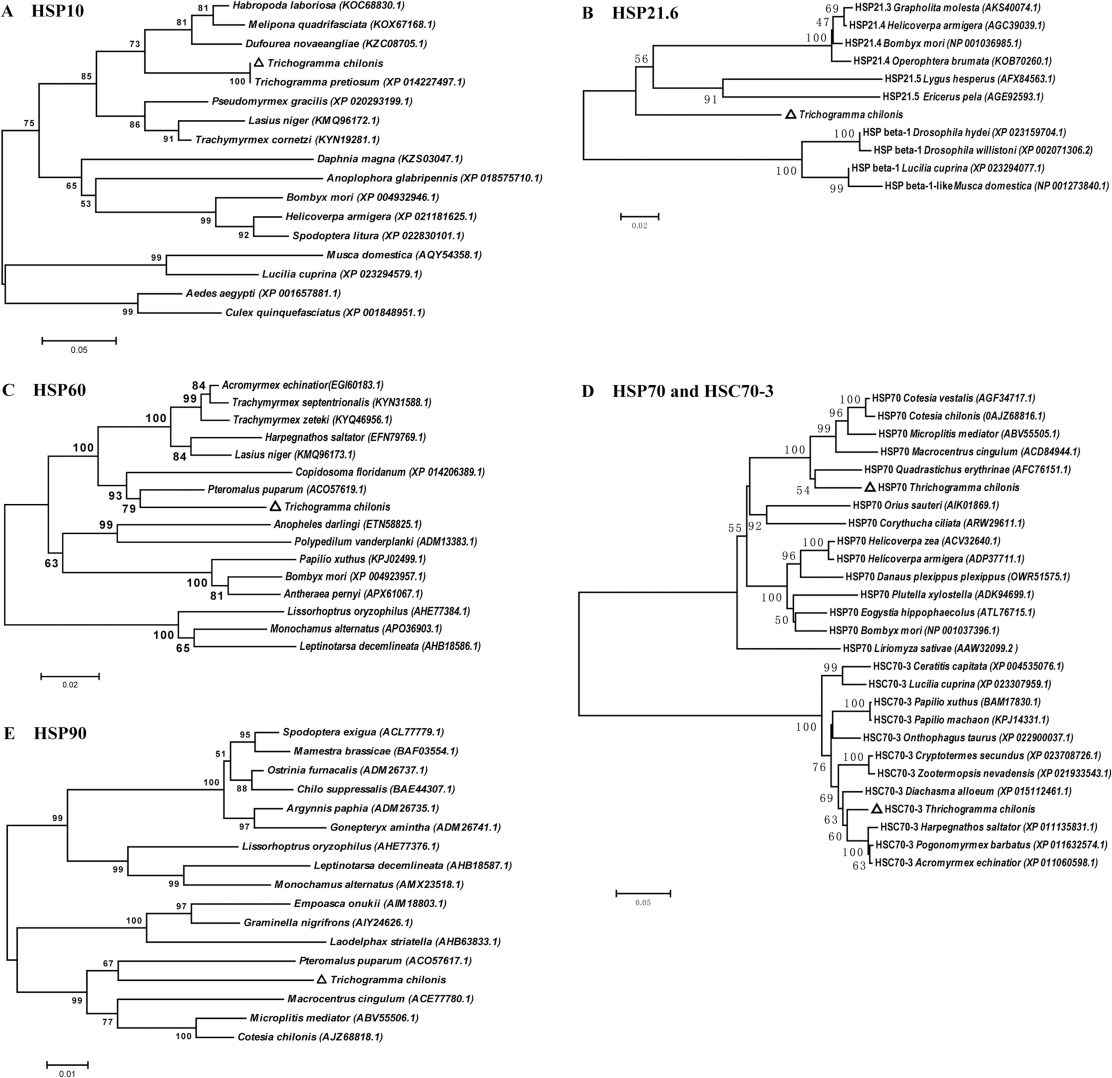
Phylogenetic analysis of TcHSPs and other homologous sequences from insects. The Neighbor-Joining (NJ) trees are constructed by using MEGA 6.0. The positions of HSPs of *Trichogramma chilonis* are marked with triangles. (A) HSP10, (B) small HSP, (C) HSP60, (D) HSP70 and HSC70-3, and (E) HSP90.

Phylogenetic trees were constructed based on the deduced amino acid sequences of *Tchsps* and their homologous sequences by the neighbor-joining method. The results revealed that HSP10 sequences from *T*. *chilonis* and *T*. *pretiosum* were clustered together into a single branch (Fig 6(A)). Two HSP60 sequences of endoparasitoid wasps (*T*. *chilonis* and *P*. *puparum*) were clustered within a branch (Fig 6(C)). A similar result was also found in the phylogenetic tree constructed with HSP90 sequences (Fig 6(E)). TcHSP21.6 showed a high similarity with HSP21.5 and HSP21.4 from other insects (Fig 6(B)). These sHSPs were clustered together and were separated from HSP beta-1 sequences. The sequences of the HSP70 family from insects presented two clusters, one with HSP70 sequences and another with HSC70-3 sequences (Fig 6(D)). The two TcHSP70 sequences showed a close relationship with their homologous sequences from the hymenopteran species.

#### Expression of *Tchsps* during development

**Fig 7:**
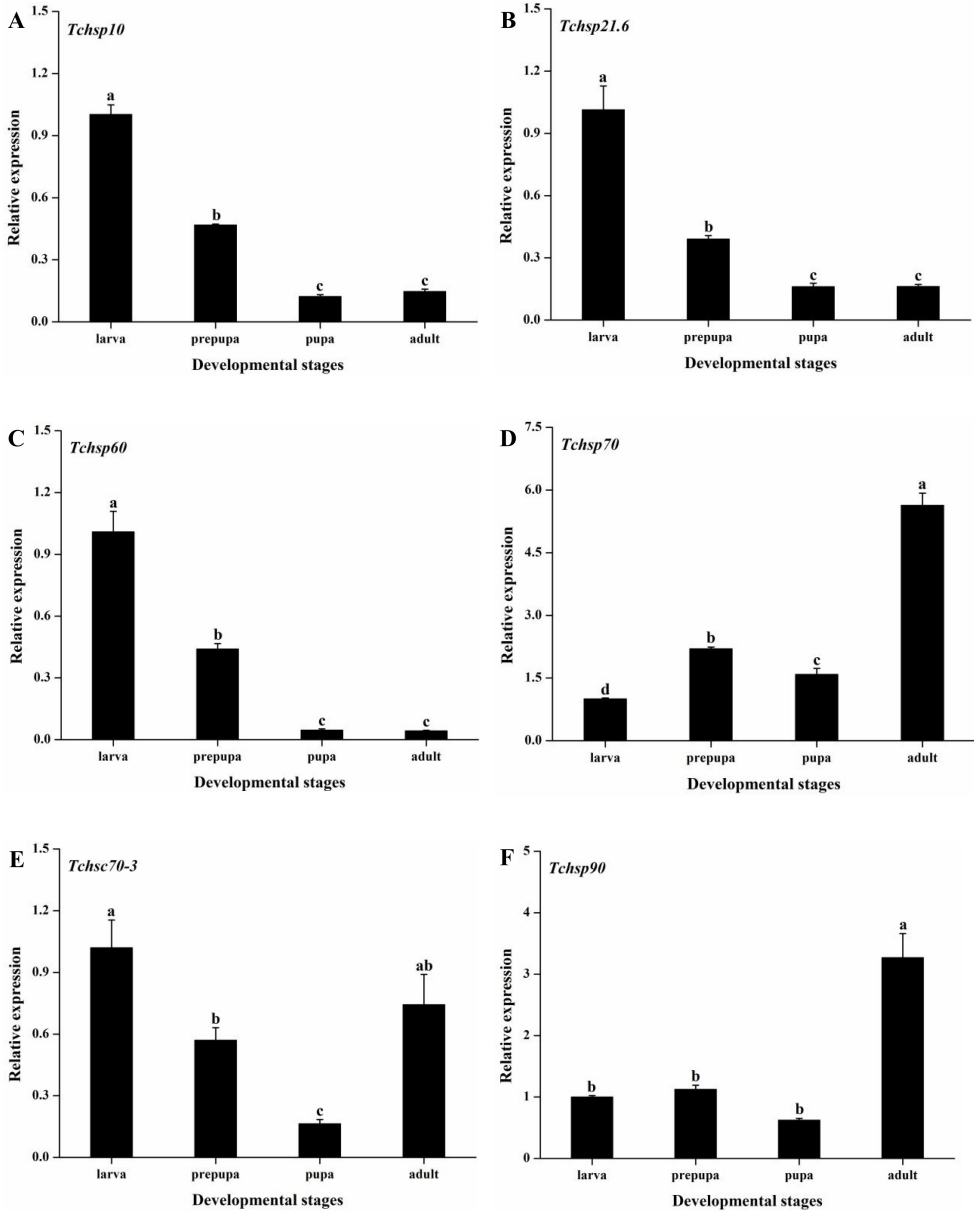
Relative expression levels of *Tchsps* during development. Data are presented as the means ± SE (n = 3). Different lowercase letters indicate significant differences. (A) *Tchsp10*, (B) *Tchsp21*.*6*, (C) *Tchsp60*, (D) *Tchsp70*, (E) *Tchsc70-3* and (F) *Tchsp90*.

Real-time PCR was used to measure the expression levels of *Tchsps* during development. The expression level in the larval stage was used as the control value. The developmental expression profiles of six *Tchsps* varied significantly in *T*. *chilonis* (*Tchsp10*: F_3, 8_ = 304.50, p < 0.001; *Tchsp21*.*6*: F_3, 8_ = 98.80, p < 0.001; *Tchsp60*: F_3, 8_ = 332.64, p < 0.001; *Tchsp70*: F_3, 8_ = 183.91, p < 0.001; *Tchsc70-3*: F_3, 8_ = 11.67, p = 0.003; *Tchsp90*: F_3, 8_ = 115.27, p < 0.001). The expression of *Tchsp10*, *Tchsp21*.*6* and *Tchsp60* decreased from the larval stage to the pupal stage and was maintained at a low level during the pupal and adult stages (Fig 7). Similarly, *Tchsc70-3* had the highest expression level during the larval stage and the lowest level during the pupal stage, while it was upregulated from the pupal stage to the adult stage. In contrast, the expression of *Tchsp70* and *Tchsp90* peaked in the adult stage, with 5.63- and 3.27-fold increases, respectively compared to the levels in the larval stage.

#### Expression profiles of *Tchsps* at different temperatures

**Fig 8:**
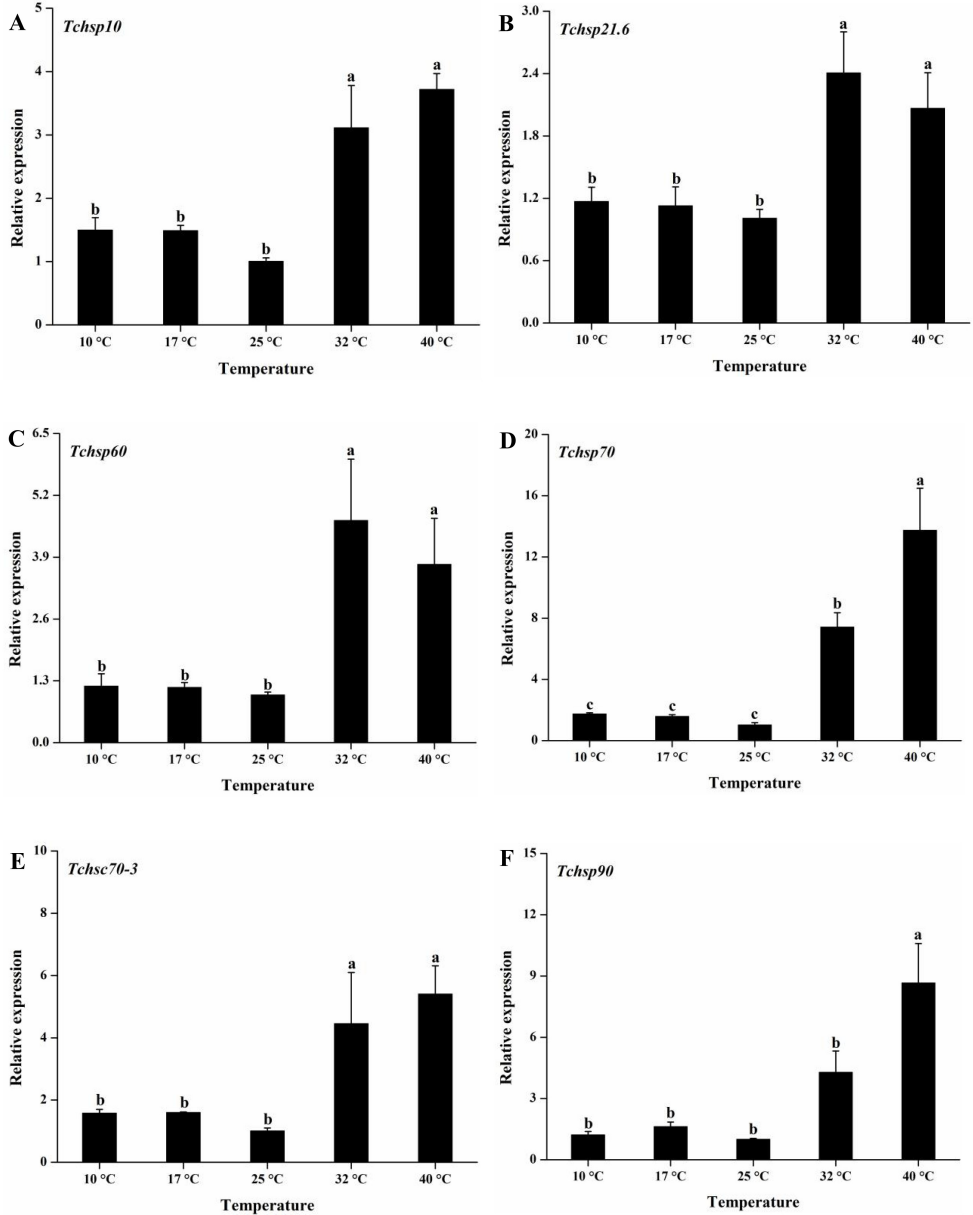
Relative expression levels of *Tchsps* after 1 h of exposure to different temperatures. Data are presented as the means ± SE (n = 3). Different lowercase letters indicate significant differences. (A) *Tchsp10*, (B) *Tchsp21*.*6*, (C) *Tchsp60*, (D) *Tchsp70*, (E) *Tchsc70-3* and (F) *Tchsp90*.

All six *Tchsps* showed the same expression pattern after exposure to different temperatures (10, 17, 25, 32 and 40°C) for 1 h (Fig 8). They were all significantly upregulated at high temperatures (32 and 40°C) compared with 25°C (*Tchsp10*: F_4, 10_ = 20.71, p < 0.001; *Tchsp21*.*6*: F_4, 10_ = 6.04, p = 0.01; *Tchsp60*: F_4, 10_ = 5.56, p = 0.013; *Tchsp70*: F_4, 10_ = 64.07, p < 0.001; *Tchsc70-3*: F_4, 10_ = 24.07, p < 0.001; *Tchsp90*: F_4, 10_ = 22.91, p < 0.001). Although the expression of *Tchsp90* at 32°C was higher than the levels at 10, 17, and 25°C, there were no significant differences. The expression levels of *Tchsp70*, *Tchsc70-3* and *Tchsp90* were significantly increased from 32 to 40°C. Among these genes, *Tchsp70* had the greatest heat response, with a 7.41 -fold increase at 32°C and a 13.74-fold increase at 40°C. On the other hand, the expression levels of six *Tchsps* slightly increased after exposure to 10 and 17°C but were not significantly different from the expression levels at 25°C.

**Fig 9:**
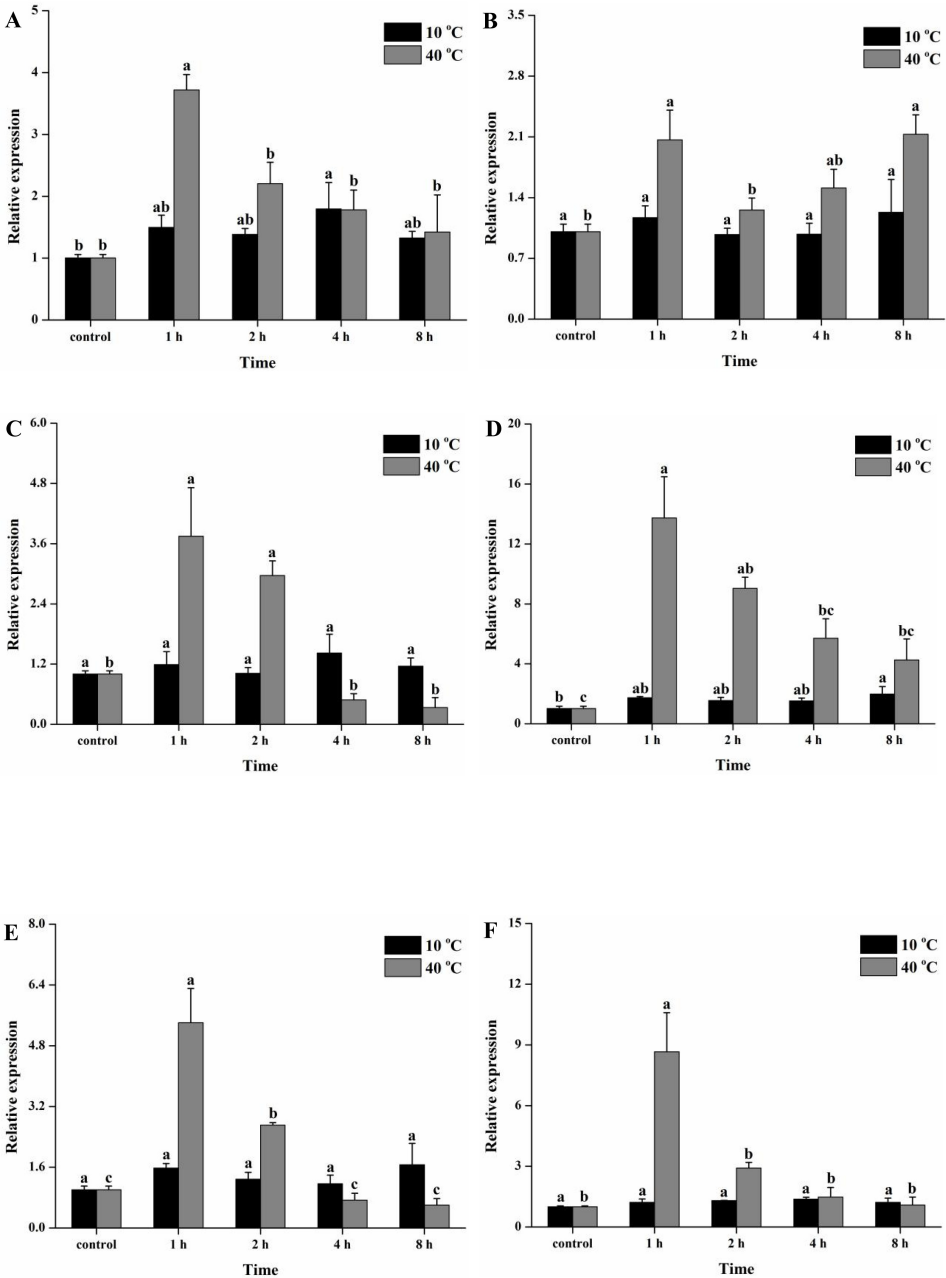
Temporal expression patterns of *Tchsps* during cold (10°C) and heat (40°C) exposure. Data are presented as the means ± SE (n = 3). Different lowercase letters indicate significant differences. (A) *Tchsp10*, (B) *Tchsp21*.*6*, (C) *Tchsp60*, (D) *Tchsp70*, (E) *Tchsc70-3* and (F) *Tchsp90*.

The temporal expression patterns of six *Tchsps* were also investigated during cold (10°C) and heat (40°C) exposure. The results indicated that the expression levels of *Tchsp10* and *Tchsp70* were significantly increased after 4 and 8 h of cold exposure (10°C), respectively (F_4, 10_ = 3.48, p ≤ 0.05; F_4, 10_ = 3.69, p = 0.043). Although the expression levels of other *Tchsps* were also slightly increased after cold exposure for different periods of time, they showed no significant differences compared with the control group (*Tchsp21*.*6*: F_4, 10_ = 0.31, p = 0.87; *Tchsp60*: F_4, 10_ = 0.49, p = 0.74; *Tchsc70-3*: F_4, 10_ = 1.37, p = 0.31; *Tchsp90*: F_4, 10_ = 1.20, p = 0.37) (Fig 9). On the other hand, the six *Tchsps* were strongly expressed after 1 h of heat exposure (40°C) (*Tchsp10*: F_4, 10_ = 10.37, p = 0.001; *Tchsp21*.*6*: F_4, 10_ = 5.08, p = 0.017; *Tchsp60*: F_4, 10_ = 11.19, p = 0.001; *Tchsp70*: F_4, 10_ = 9.98, p = 0.002; *Tchsc70-3*: F_4, 10_ = 23.38, p < 0.001; *Tchsp90*: F_4, 10_ = 12.25, p = 0.001). The expression of *Tchsp10*, *Tchsp60*, *Tchsp70*, *Tchsp70-3* and *Tchsp90* decreased from 1 h to 8 h. However, *Tchsp21*.*6* exhibited the highest levels at 1 h and 8 h.

## Discussion

In this study, full-length cDNAs of six HSP genes were obtained from *T*. *chiloni*s, including *Tchsp10*, *Tchsp21*.*6*, *Tchsp60*, *Tchsp70*, *Tchsc70-3* and *Tchsp90*. TcHSP10 contained a mobile loop, which is consistent with the characteristic of other HSP10 sequences described in many studies [31–33]. Through the mobile loop, HSP10 interacts with the HSP60, which helps the folding of protein [34, 35]. *Tchsp21*.*6* encoded a polypeptide of 191 amino acids with a predicted molecular weight of 21.68 kDa. The deduced amino acid sequence of *Tchsp21*.*6* contained an α-crystallin domain (ACD), which is a characteristic feature of the sHSPs family [36, 37]. This family is composed of many members containing variable N- and C-terminal extensions [32, 38]. TcHSP60 belongs to the mitochondrial HSP60 family, containing a conserved signature motif (AAVEEGIVPGGG) and a GGM motif [23, 39]. The GGM motif at the C-terminus has been suggested to provide a suitable physical environment for protein folding [40]. The ATP/ADP binding sites were found in TcHSP60, which have also been identified in HSP60 sequences from *Rhopalosiphum padi* (L.) (Homoptera: Aphididae) and *Lucilia cuprina* (Diptera: Calliphoridae) [39, 41]. The highly conserved motif among HSP60 sequences may indicate that a similar mechanism of coupling ATP hydrolysis to the substrate-refolding process exists [14, 39, 42]. The two TcHSP70 sequences had three conserved HSP70 family signatures and a non-organellar consensus motif, in accordance with the structures of the HSP70 sequences described in *Nilaparvata lugens* (Homoptera: Delphacidae), *Sitodiplosis mosellana* (Diptera: Cecidomyiidae) and *Habrobracon hebetor* (Hymenoptera: Braconidae) [43–45]. The two TcHSP70 sequences showed high similarity with their homologous sequences from other insects. These results indicated that the members of HSP70 family are highly conserved [46].

It has been well reported that HSPs are involved in the development of insects [47, 48]. However, the expression patterns of *hsps* during development vary in insects [49, 50]. For example, the expression of *hsp60* increases from the larval stage to the adult stage in *Liriomyza sativa* (Diptera: Agromyzidae), while it decreases from nymph to adult in *R*. *padi* [39, 47]. In this study, *Tchsp10* and *Tchsp60* levels decreased during development, which is consistent with the findings of report on *Galeruca daurica* (Coleoptera: Chrysomelidae) [50]. Moreover, *Tchsp21*.*6* showed the same expression pattern as *Tchsp10* and *Tchsp60*. The high expression of three *Tchsps* in the larval stage indicated that these genes may be related to larval development. In *T*. *chilonis*, *Tchsp70* and *Tchsp90* levels peaked at the adult stage, and *Tchsc70-3* expression was upregulated from the pupal stage to the adult stage. It is different from the findings in *S*. *exigua* and *Frankliniella occidentalis* (Thysanoptera: Thripidae) [48, 49]. In contrast, in the endoparasitoid wasp *M*. *cingulum*, *hsp70* and *hsp90* are highly expressed in the pupal and adult stages [26]. The high expression of *hsp70* begins at the third-instar larval stage, when *C*. *vestalis* comes out of the host [27]. As an egg endoparasitoid, adults of *T*. *chilonis* emerge from host, thus facing very different environmental stresses. These HSP genes, i.e., *Tchsp70*, *Tchsp90* and *Tchsc70-3*, might be needed to overcome these challenges.

HSPs also play important roles in the response to temperature stress [51]. As described in the introduction, HSPs are molecular chaperones that help to prevent potential damage to cellular and molecular structures under temperature and other stresses [15, 52]. Altered expression patterns of *hsps* have been widely reported under temperature stresses, although these responses seem to be species-specific among insects [39, 43]. In *T*. *pui*, the expression of *hsp90*, rather than *hsp7*0, changes in response to temperature [22]. However, *hsp90* and *hsp70* in *Empoasca onukii* (Hemiptera: Cicadellidae) are both highly expressed under cold and heat treatments [53]. *Cchsp60* in *C*. *chilonis* responds to cold stress but is insensitive to heat stress [24]. In contrast, the highest expression level of *hsp6*0 in *P*. *puparum* appears at 36°C [23]. In this study, *Tchsps* were sensitive to high temperatures (32 and 40°C), which is consistent with the expression patterns of *hsps* observed in other species [23, 48, 50]. The expression of *Tchsp10*, *Tchsp21*.*6* and *Tchsp60* showed the same expression pattern in response to high temperatures, each being significantly upregulated under high temperatures but with no significant differences at 32 and 40°C. The upregulation of *hsp10* and *hsp60* has also been reported in *Apostichopus japonicus* (Echinodermata: Holothuroidea) and *G*. *daurica* [31, 50]. HSP10 is considered the co-chaperone of HSP60 and interacts with HSP60 in the same heat shock pathway [33, 54]. TcHSP21.6 belongs to the sHSPs family and showed high similarity with HSP21.5 and HSP21.4, which have been proven to respond to heat stress in *E*. *pela* female adults and in *B*. *mori* [55, 56]. *Tchsp70* exhibited the highest expression at 40°C. TcHSC70-3 is a constitutively expressed protein that is also induced by heat. These results agreed with the reports that the HSP70 family includes the major heat shock proteins induced by thermal stresses [24, 26, 48]. *Tchsp90* exhibited the same expression pattern as the two TcHSP70 genes, being significantly upregulated from 32 to 40°C. Similar response patterns have been reported in *E*. *onukii*, *S*. *exigua* and two *Liriomyza* species [21, 48, 53]. The upregulation of *Tchsps* expression in *T*. *chilonis* may contribute to the improvement of thermal tolerance. Some reports have documented that pupae of *T*. *chilonis* can endure a high temperature of 40°C [57, 58]. On the other hand, the heat-responsive expression of *Tchsps* also implies that the expression of *Tchsps* could be a potential indicator of the heat stress response [8, 16]. Further study is needed to support this speculation. Cold treatments (10 and 17°C) for 1 h led to a slight, but not significant, increase in the expression of all *Tchsps*, indicating that *Tchsps* are insensitive to low temperatures. In different insects, the expression patterns of these genes vary under cold exposure. For instance, *hsp60* cannot be induced by cold in two *Liriomyza* species but could be upregulated in response to low temperature in *C*. *chilonis* [21, 24]. The expression of *hsp70* decreases in response to cold shock in *N*. *lugens* while it increases in *E*. *onukii* [43, 53]. Some reports have indicated that the recovery from cold, rather than the direct cold stress, triggers the high expression of HSP genes [49, 59].

Under the 40°C treatment, the expression of all *Tchsps* exhibited a time-dependent response. Except for the expression level of *Tchsp21*.*6* at 8 h, expression levels of these genes significantly increased after 1 h of exposure to 40°C and dramatically decreased at subsequent time points. This result indicated that the induction of *hsps* may be rapid and transitory. This phenomenon has also been reported in numerous studies [21, 31, 44]. Many studies have attributed the decrease in *hsp* expression to the energy balance and metabolic disorder [50]. In addition, previous studies have speculated that the synthesis of HSPs requires excess energy consumption, imposing stresses on various metabolic activities [60, 61]. The activity of enzymes is restricted under long-term heat exposure, which may also result in the decrease of *hsp* expression [62]. Under the 10°C treatment, the expression of *Tchsp21*.*6*, *Tchsp60*, *Tchsc70-3* and *Tchsp90* did not dramatically change at any time points. *Tchsp10* and *Tchsp70* exhibited a low-intensity cold response at 4 and 8 h. This result suggested that *Tchsps* have low or no sensitivity to cold temperatures.

## Conclusions

In summary, six *Tchsps* were cloned and characterized from *T*. *chilonis*, namely, *Tchsp10*, *Tchsp21*.*6*, *Tchsp60*, *Tchsp70*, *Tchsc70-3* and *Tchsp90*. These *Tchsps* exhibited different expression profiles at different developmental stages, suggesting they may be involved in the development of *T*. *chilonis*. In pupae of *T*. *chilonis*, the expression profiles of these genes could be induced by heat shocks (32 and 40°C for 1 h) but did not change in response to cold shocks (10 and 17°C for 1 h). In addition, their expression levels showed time-dependent responses to heat exposure. *Tchsp10* and *Tchsp70* exhibited a low-intensity cold response at 4 and 8 h. However, *Tchsp21*.*6*, *Tchsp60*, *Tchsc70-3* and *Tchsp90* did not respond to cold exposure. Due to the difficultly of sampling caused by the tiny size and parasitic characteristics of *T*. *chilonis*, our study initially explored the expression patterns of *hsps* during development and temperature stresses. Our study may aid in a better understanding of the roles of *hsps* at different development stages and in response to temperature stresses.

## Supporting information

S1 Table Primers used for different PCRs. (DOCX)
