# Supplementary material for: Molecular characterization and expression of six heat shock protein genes in relation to development and temperature in Trichogramma chilonis
Source: PLoS One. 2018 Sep 18;13(9):e0203904. doi: 10.1371/journal.pone.0203904 (PMC6143235; doi:10.1371/journal.pone.0203904)
Supplement: S1 Table — (DOCX) [file pone.0203904.s001.docx]

S1 Table. Primers for cDNA cloning and real time quantitative PCR.

| Gene | Method | Primer name | Primer sequence (5’→ 3’) |
| --- | --- | --- | --- |
| Tchsp10 | PCR | hsp10MF | CCGCAGTGCAATTCGTCG |
|  |  | hsp10MR | TGTTTATTCCTCCAACTTAGCC |
|  | 3’ RACE | hsp10F1 | ATGTCTGCTGCCGCTGTTGTTA |
|  |  | hsp10F2 | ATCAAGGTTGGCGATGTAGTGTT |
|  | 5’ RACE | hsp10R1 | TAACAACAGCGGCAGCAGACAT |
|  |  | hsp10R2 | TAACAGCTTCTGCCCTTTGGATA |
|  | Real-time qPCR | Qhsp10F | TTATCCAAAGGGCAGAAGC |
|  |  | Qhsp10R | GTAACACTACATCGCCAACCT |
| Tchsp21.6 | PCR | hsp21.6MF | AACATACAACAGTGCGTCAGTC |
|  |  | hsp21.6MR | CAGTCACAGGTAGCGATAACAT |
|  | 3’ RACE | hsp21.6F1 | TCCGTCTACAGGGAATACAACCG |
|  |  | hsp21.6F2 | CCAAAGGCACGAATCCCGAGACC |
|  | 5’ RACE | hsp21.6R1 | TTTCGTGGTTCTGCGGGCTCA |
|  |  | hsp21.6R2 | GGTCTCGGGATTCGTGCCTTTGG |
|  | Real-time qPCR | Qhsp21.6F | ACCTGGTTGGATGGCCTCA |
|  |  | Qhsp21.6R | TTGTATTCCCTGTAGACGGATTTT |
| Tchsp60 | PCR | hsp60MF1 | CTTTAGGTAACTCGGTAACAACGG |
|  |  | hsp60MR1 | GGATGTGAGATTTGGAGCAGAA |
|  |  | hsp60MF2 | GATTTGGAGCAGAAGTTAGAGC |
|  |  | hsp60MR2 | GGTAGCCTTTCGGTTGTCTC |
|  | 3’ RACE | hsp60F1 | GAGTAGGTGGAAGCAGCGAAGT |
|  |  | hsp60F2 | TGCCTTGCTTACAAATCGCTCAG |
|  | 5’ RACE | hsp60R1 | ATCACAAGTGGTTTACGCTGAGAAT |
|  |  | hsp60R2 | GACCCTTAGGACCCATAGTCACAGC |
|  | Real-time qPCR | Qhsp60F | CATAATCCCTGCTCTTGAACTTGC |
|  |  | Qhsp60R | TGGTAGCCTTTCGGTTGTCTC |
| Tchsp70 | PCR | hsp70MF1 | GCTTGTACTCGTCCTTCTCGGCTAA |
|  |  | hsp70MR1 | CAGACGCAAACCTTCACGACCTAC |
|  |  | hsp70MF2 | CGCCGTCATCACAGTACCCG |
|  |  | hsp70MR2 | ACCACCCGTCCATCAAACAAAC |
|  | 3’ RACE | hsp70F1 | CAACAACATCCGCATCACCAAC |
|  |  | hsp70F2 | CGCATCACCAACGACAAAGGC |
|  | 5’ RACE | hsp70R1 | CACCACCCAAGTCGAAGATGAGG |
|  |  | hsp70R2 | TTGATGATTCTGAGTACGTTGAGGC |
|  | Real-time qPCR | Qhsp70F | ACAACAACACCTTAGCCGAGAA |
|  |  | Qhsp70R | ACTGCTGTCCACATCCTTGAG |
| Tchsc70-3 | PCR | hsc70-3MF | GTCACCGTGCCCGCTTACTT |
|  |  | hsc70-3MR | CTGGACGATGTCTGTCAGTTCTTTCT |
|  | 3’ RACE | hsc70-3F1 | TCCACCGCCTCCGACAACCA |
|  |  | hsc70-3F2 | CCATCCAGGTCTACGAGGGTGAGC |
|  | 5’ RACE | hsc70-3R1 | GTAAGCGATGGCAGCGGCAGTTGG |
|  |  | hsc70-3R2 | GTTGTCGGAGGCGGTGGAGA |
|  | Real-time qPCR | Qhsc70-3F | CTCAAAGACAGGCCACTAAAGACG |
|  |  | Qhsc70-3R | CGCCCAAATCGAAGACAAGCAC |
| Tchsp90 | PCR | hsp90MF | GAAACTGCCGAAGAGGTTGAGAC |
|  |  | hsp90MR | ATGTTAGCGGTCCAGCCATACTG |
|  | 3’ RACE | hsp90F1 | CCACGAAGACAGCAGCAACC |
|  |  | hsp90F2 | TTTGGAGTTGCCTTTGGATG |
|  | 5’ RACE | hsp90R1 | GTGTCAATGATGGTGAGGGTGC |
|  |  | hsp90R2 | GGTTGTTTACCAGGTCGGCTTT |
|  | Real-time qPCR | Qhsp90F | GAAATCAACCCAGACCATCCAG |
|  |  | Qhsp90R | GCTCATCAAGAGTGAAGCCAGA |
| *gadph* | Real-time qPCR | Q*gadphF* | AAAACGCAACACTCCCAGCT |
|  |  | Q*gadphR* | CCCGTGTCATCGACCTCATC |
